# Early Behavioural Signs among Nepalese Children with Autism Spectrum Disorder: A Descriptive Cross-sectional Study

**DOI:** 10.31729/jnma.8499

**Published:** 2024-03-31

**Authors:** Manju Shrestha, Rinkal Suwal, Niranjan Thapa, Suchit Thapa Chhetri, Bishal Kunwor, Shiva Kumar Regmi, Oshan Shrestha, Bipin Mehta

**Affiliations:** 1 Department of Paediatrics and Adolescent Health, Centre For Autism, Baluwatar, Kathmandu, Nepal; 2 B.P. Eye Foundation, Lokanthali, Bhaktapur, Nepal; 3 Nepalese Army Institute of Health Sciences, Sanobharyang, Kathmandu

**Keywords:** *autism spectrum disorder*, *behavior*, *children*, *language*, *sleep*

## Abstract

**Introduction:**

Autism Spectrum Disorder is a complex neurodevelopmental condition. Early identification of symptoms is crucial for timely intervention, yet diagnosing very young children can be challenging due to the variability in symptom presentation and the influence of other developmental factors. This study aimed to find the prevalence of the emergence of early behavioural signs in Nepalese children with Autism Spectrum Disorder.

**Methods:**

A descriptive cross-sectional study was conducted at the Centre for Autism in Kathmandu, Nepal, from January 2023 to June 2023. Ethical approval was obtained, and a sample of 120 children diagnosed with Autism Spectrum Disorder was included in the study. Convenience sampling method was used. Point estimate at 95% Confidence Interval was calculated.

**Results:**

Among 120 children with Autism Spectrum Disorder, the prevalence of emergence of early behavioural signs was seen in 112 (93.33%) (88.83-97.77, 95% Confidence Interval) children.

**Conclusions:**

This study provides insights into the emergence of early behavioural signs in Nepalese children with Autism Spectrum Disorder which align with global patterns in prevalence and severity.

## INTRODUCTION

Autism spectrum disorder (ASD) is characterized by impairment in social/communication along with repetitive, restricted, and stereotyped behaviors.^1^ The etiology is multifactorial, originating from a complex interplay between genetic and environmental factors.^2-4^ The presentation varies in range and severity and often changes with the acquisition of other developmental skills.^5,6^ The various signs and symptoms of ASD begin to emerge in the first year of life.^1,7^

Early detection of ASD is considered difficult, especially in very young children, as characteristic behaviors may be hard to identify until they become evident and separable from general developmental delay.^8,9^ Furthermore, symptoms and behaviors may differ between individual cases, age groups and genders and the development of motor skills is rarely affected.^10^

This study aimed to find the prevalence of the emergence of early behavioural signs in Nepalese children with ASD.

## METHODS

This descriptive cross-sectional study was conducted among the children in the Centre for Autism (CFA), present in Kathmandu, Nepal from January 2023 to June 2023 after obtaining ethical approval from the Nepal Health Research Council (Reg. No. 2383). Individual children with ASD presenting in the Centre of Autism were considered as sampling units. A convenience sampling method was used. The sample size was calculated using the following formula:


$$\begin{array}{ll} \text{n} & ={\text{Z}}^{2}\times \frac{\text{p}\times \text{q}}{{\text{e}}^{\text{2}}}\\ & =1.96^{2}\times \frac{0.50\times 0.50}{0.08^{2}}\\ & =151 \end{array}$$

Where,

n = minimum required sample size
Z = 1.96 at 95% Confidence Interval (CI)
p = prevalence taken as 50% for maximum sample size
q = 1-p
e = margin of error, 8%

The calculated sample size for our study was 151. Children with a history suggestive of ASD aged five years or younger, and diagnosed with the Autism Diagnostic Observation Schedule (ADOS) and whose parents provided consent were included in the study. On the other hand, the exclusion criteria included children with psychiatric disorders, those receiving medication for malignant diseases, children older than five years, and caregivers unwilling to give consent. The questionnaire composed of three parts. The first part (ASD children) included inquiry regarding general information and socio-demographic status. The second part of the questionnaire included the core symptoms of children with ASD which was evaluated by using Childhood Autism Rating Scale (CARS) and Autism Diagnostic Observation Schedule-Second Edition (ADOS). Third part consisted of early behavioural symptoms based on grading of severity of ASD.

Participants were evaluated based on Social interaction and Relationships, Language disorder, Stereotyped behaviors and activities, Motor skills, and Regulation: Feeding/Sleep disorders. Each of these conditions have multiple behavioural points which were recorded and rated by examiners. Data was entered into Microsoft Excel 2007 and analysed. Point estimate at 95% CI was calculated.

## RESULTS

A total of 151 cases diagnosed children with ASD were taken and analyzed. Among them only 120 children with ASD were included in the study. Among 120 children with ASD, the prevalence of emergence of early behavioural signs was seen in 112 (93.33%) (88.83-97.77, 95% Confidence Interval) children.

**Figure 1:**
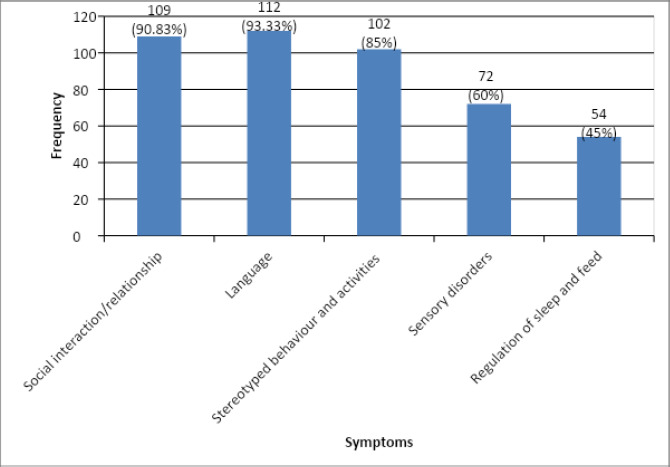
Categorical variation of the early Symptoms of ASD.

Our study showed that among 120 patients diagnosed with ASD, 109 (90.83%) of children had early emergence of symptoms that were seen with social interaction and relationship. Among all these variables regulation of sleep and feed was found in only 54 (45%) of the children as an early symptom of ASD (Figure 1).

**Figure 2:**
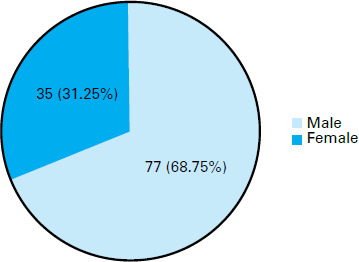
Sex distribution of children with ASD showing early behavioural signs.

A total of 38 (33.92%) were between 36 - 48 months of age followed by 37 (33.04%) above 48 months of age (Figure 2).

**Figure 3:**
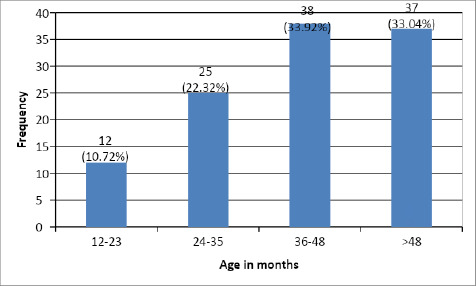
Age group of children with early behavorial signs in ASD.

A total of 37 (33.14%) children with early behavioural signs belonged to the age group between 48 and above 48 months followed by 36-48 months (Figure 3).

**Figure 4:**
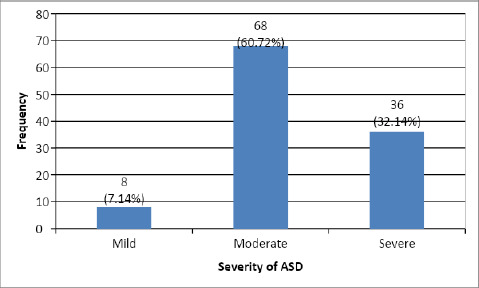
Severity of ASD among children with early behavioural signs.

Among the children with early behavorial signs, 68 (60.7%) children with ASD in our study had moderate levels of ASD followed by 36 (32.14%) with severe levels and 8 (7.14%) had mild levels of ASD (Figure 4).

**Figure 5:**
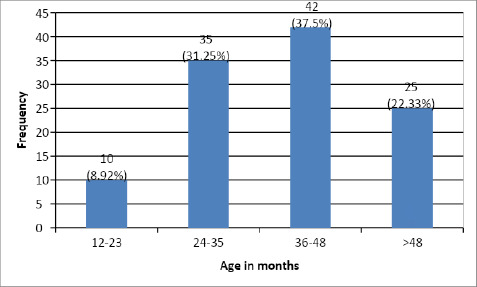
Age at onset of ASD among children with early behavorial signs.

Out of 112 children, 42 (37.50%) children showed the symptoms at the age of 36-48 months. Only 10 (8.92%) of children showed emergence of early symptoms at 12-23 months (Figure 5).

## DISCUSSION

The study aimed to investigate the prevalence of early behavioural signs in Nepalese children with ASD. The findings reveal that among the 120 children diagnosed with ASD, 93.33% exhibited early behavioural signs. The most common early symptoms were related to language regression, with 90.8% of children experiencing such issues. Social interaction and relationship difficulties were also prominent, affecting 93.33% of the children. Sleep and feeding regulation problems were observed in 45% of the cases.

There is still debate as to what is the most effective strategy for identifying the early signs of autism in very young children. The American Academy of Paediatrics and the American Academy of Neurology and Child Neurology have advocated screening for autism in all children between the ages of 18 and 24 months; evidence suggests that uptake of these recommendations has been limited.^1,11^ Our study involved a comprehensive assessment of 120 children diagnosed with ASD utilizing the rigorous criteria provided by the Autism Diagnostic Observation Schedule (ADOS) and Clinical Assessment of Autism Related Behaviors (CARE) to assess for the early symptoms of ASD and its onset.

Regarding categorical variability, our study findings indicate that an overwhelming majority (93.33%) of the children exhibited early symptoms related to language regression, highlighting the prominence of these indicators in diagnosing ASD. Notably, a significant number of children with ASD (90.83%) experienced symptoms related to social interaction and relationships as one of their initial symptoms, emphasizing its significance as a potential early marker. In contrast, sleep and feeding regulation issues were observed in a comparatively lower proportion, affecting only 45% of the children with ASD in our sample. A study by Hansen et al. showed that a greater percentage of children exhibited social losses (46%) as compared to language skills (18%).^12^ Similarly, another study by Goldberg et al. showed that 38% had social-only regression and 5% had language-only regression.^13^ By elucidating these different patterns of regression, the studies shed light on the heterogeneity of ASD and the varied ways in which children may manifest developmental regression. Such insights are crucial for enhancing early identification, intervention, and support for children with ASD and their families.

Within our study, a substantial proportion of children diagnosed with ASD exhibited early symptoms between 36-48 months, accounting for 37.5% of the studied population. Following closely, the age group of 24-35 months displayed early symptoms in 31.25% of the children. In contrast, a relatively smaller percentage (8.92%) of children displayed the emergence of early symptoms within the age range of 12-23 months. In contrast to the findings of the cross-sectional study conducted by Parmeggiani et al,. our study yielded divergent results.^14^ Specifically, their study identified a notably higher percentage (41.9%) of children who exhibited early symptoms of autism between the ages of 7 and 12 months, while a mere 1.9% displayed such symptoms between the ages of 37 and 51 months. These discrepant findings highlights the potential variability in the age of symptom onset within the population of children diagnosed with ASD.

In terms of the distribution of cases by sex, our study found male predominance (68.75%) among the participants, consistent with previous research on ASD prevalence. This aligns with a study conducted by Baio et al, which reported a higher prevalence of ASD in males.^4^ The consistency in gender distribution strengthens the notion of a male vulnerability factor in ASD across different populations. The severity levels of ASD identified in our study showed that the majority of children (60.72%) exhibited a moderate level of impairment, followed by a considerable proportion (32.14%) with severe ASD. These findings are consistent with studies which explored severity levels and maladaptive behaviors.^15^ This similarity suggests that the ASD symptoms in Nepalese children aligns with the broader global spectrum.

While our study contributes to understanding the emergence of early behavioural signs in Nepalese children with ASD, it is important to acknowledge its limitations. The study's limitations include a restricted sample size from a specific geographic location, a cross-sectional design hindering causal inferences, potential subjectivity in diagnostic tools, and a lack of exploration into cultural influences. These factors may impact the generalizability of findings, suggesting the need for larger, diverse samples, longitudinal studies, and investigations into cultural and linguistic nuances for a more comprehensive understanding of early behavioural signs in Nepalese children with ASD.

## CONCLUSIONS

This study highlights the male predominance, delayed diagnosis, severity distribution and categorical variability of regression of different characters in ASD among Nepalese children. The findings align with global patterns of ASD prevalence and severity but the age and pattern of early symptoms which appeared showed variability. The findings advocate for heightened awareness campaigns and culturally sensitive interventions, urging future research to develop and assess the impact of such strategies in the Nepalese context.
